# Integration of Ultra-Low Volume Pneumatic Microfluidics with a Three-Dimensional Electrode Network for On-Chip Biochemical Sensing

**DOI:** 10.3390/mi12070762

**Published:** 2021-06-28

**Authors:** Saurabh Tomar, Charlotte Lasne, Sylvain Barraud, Thomas Ernst, Carlotta Guiducci

**Affiliations:** 1 Laboratory of Life Sciences Electronics, École Polytechnique Fédérale de Lausanne, 1015 Lausanne, Switzerland; charlotte.lasne@gmail.com (C.L.); carlotta.guiducci@epfl.ch (C.G.); 2 Laboratoire d’Électronique et de Technologie de l’Information (LETI), Commissariat à l’Énergie Atomique et aux Énergies Alternatives (CEA), CEDEX 9, 38054 Grenoble, France; sylvain.barraud@cea.fr (S.B.); thomas.ernst@cea.fr (T.E.)

**Keywords:** SU-8, reference electrode, packaging, PDMS, microfluidics, ISFET, post-CMOS processing, pneumatic valves, flexible electronics

## Abstract

This paper reports a novel miniaturized pseudo reference electrode (RE) design for biasing Ion Sensitive Field Effect Transistors (ISFETs). It eliminates the need for post-CMOS processing and can scale up in numbers with the CMOS scaling. The presented design employs silane-mediated transfer of patterned gold electrode lines onto PDMS microfluidics such that the gold conformally coats the inside of microfluidic channel. Access to this electrode network is made possible by using “through-PDMS-vias” (TPV), which consist of high metal-coated SU-8 pillars manufactured by a novel process that employs a patterned positive resist layer as SU-8 adhesion depressor. When integrated with pneumatic valves, TPV and pseudo-RE network were able to bias 1.5 nanoliters (nL) of isolated electrolyte volumes. We present a detailed characterization of our pseudo-RE design demonstrating ISFET operation and its DC characterization. The stability of pseudo-RE is investigated by measuring open circuit potential (OCP) against a commercial Ag/AgCl reference electrode.

## 1. Introduction

In recent times, Ion Sensitive Field Effect Transistors (ISFETs) have rightfully harnessed the technological advances of CMOS manufacturing for applications in biosensing. ISFETs have been scaled down to as low as 10 nm wide [1] and scaled up to hundreds of millions of transistors per chip [2]. As evident, much research effort has been spent on the silicon part of biosensing; however, the microfluidic part has comparatively lagged behind. State-of-the-art ISFET biosensing solutions still utilize big electrolyte pools with macroscopic reference electrodes for biosensing [1,3,4,5]. Even when microchannels are employed, they still use an external macroscopic reference electrode [6]. Moreover, such designs lack the ability to multiplex different reactions on the chip. Due to the use of macroscopic RE, the microfluidic cavity can accommodate the monitoring of only one reaction at a given time. On-CMOS miniaturized reference electrodes have addressed some of these limitations [3]. However, such solutions require post-fabrication processing of CMOS chips. As post-CMOS processing is a nonstandard process, it may introduce variabilities in the sensing layer, which can affect chip-to-chip response.

To truly achieve the potential of CMOS technology for biosensing implies to be able to simultaneously carry out hundreds or thousands of biosensing reactions in mutually and electrolytically isolated, micro-sized biochemical incubation chambers, each of which is monitored in real-time by one or more ISFET sensor. Such approach requires parcellization strategies in microfluidics that can integrate a miniaturized reference electrode and all the while avoiding any post-CMOS processing. Here, we propose a microfluidic design that successfully addresses all the above issues.

**Figure 1:**
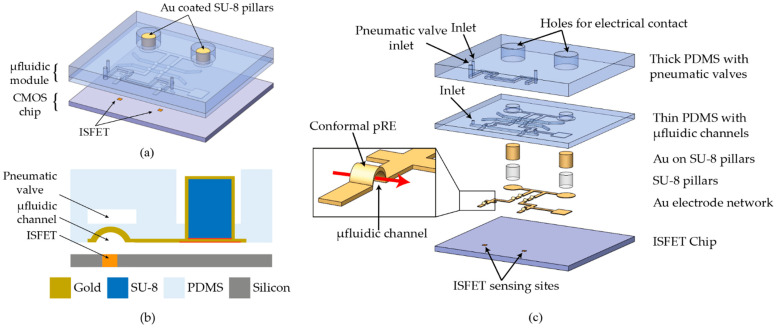
(**a**) 3D schematic of the microfluidic module on a CMOS chip consisting of two nanoliter sized chambers that are created upon actuation of a pneumatic valve. (**b**) Cross-sectional schematic of the module showing the metal-coated “through-PDMS-vias”. (**c**) Exploded view of the microfluidic module showing the comprising layers. Inset shows conformal pseudo-RE with liquid flow (red arrow) in the microchannel.

In this paper, we present a non-planar miniaturized pseudo reference electrode (pseudo-RE) that conformally coats the inside of the PDMS microfluidic channel. The design concept is presented in Figure 1 and Figure S3. The pseudo-RE network is integrated into PDMS and is electrically accessible by “through-PDMS-vias” (TPV). TPV are made of gold-coated, very high SU-8 pillars that go through the PDMS matrix. The presented strategy allows reversible integration of a miniaturized reference electrode close to the ISFET sensing surface. Reaction mixture parcellization into nanoliter (nL) size volumes is achieved by integration of pneumatic valves. These electrolytically isolated parcels over ISFETs are biased by an “in-chamber” pseudo-RE, thereby enabling multi-reaction sensing on a single chip. The need for post-CMOS processing chips is eliminated by the combination of conformal pseudo-RE in microfluidics and TPV.

## 2. Materials and Methods

### 2.1. Microfabrication Process

**Figure 2:**
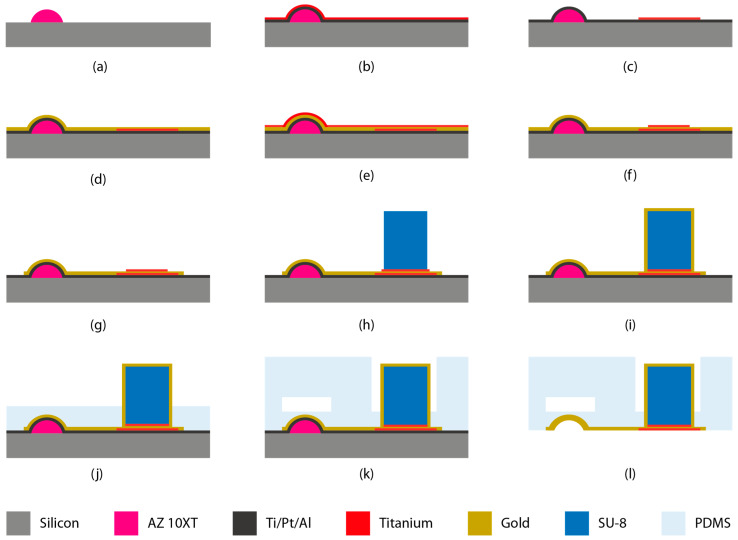
(**a**–**l**) Wafer level process steps for fabrication of the microfluidic module featuring pseudo-RE and TPV.

The whole process flow was carried out on a 4-inch silicon wafer. After priming the silicon wafer with HMDS (hexamethyldisilazane), a 10 µm of positive tone resist (AZ 10XT) was spin-coated over it. A negative mold for 150 µm wide microfluidic flow channels was patterned by standard photolithography of AZ 10XT resist. Patterned AZ 10XT was then baked at 140 °C (ramping rate of 4 °C/min) for 2 h to reflow the resist in order to achieve a rounded cross-section (Figure 2a) for microfluidic channel mold from an initially rectangular one. The reflow of the resist also leads to an increase in the channel mold height from 10 µm to 12 µm at its highest point. To protect the resist mold from subsequent processing steps, the whole wafer was passivated by bilayer evaporation (Leybold Optics Lab 600H, Bühler Leybold Optics, Alzenau, Germany) of Ti/Pt (10/100 nm). A 500 nm of sacrificial aluminum layer was sputtered (Pfeiffer SPIDER 600, Pfeiffer Vacuum, Asslar, Germany) on top of Platinum (Figure 2b). The aluminum layer is later etched away via anodic dissolution to release the final microfluidic module from the silicon wafer.

A 20 nm adhesion layer of Titanium (Ti(1)) was evaporated on top of aluminum. The evaporated Titanium was then spin-coated with 5 µm AZ ECI 3027 positive tone resist. The resist was UV exposed on mask aligner (KarlSüss MA6/BA6) with a dose of 415 mJ/cm^2^. The resist was developed in AZ Developer MIC (Microchemicals GmbH, Ulm, Germany). Titanium (Ti) was etched by ion beam etching (Veeco Nexus IBE 350, Veeco, Plainview, NY, USA) to define Ti regions, which promote adhesion for gold (Figure 2c). After stripping the resist in oxygen plasma (Tepla GiGAbatch, PVA TePla AG, Wettenberg, Germany), a 200 nm of gold layer (Au(1)) was sputtered (Alliance-Concept DP650, Annecy, France) to cover the entire wafer (Figure 2d). Subsequently, a second 20 nm adhesion layer of titanium (Ti(2)) was evaporated on top of gold (Figure 2e). Just like the former titanium layer, the second layer (Ti(2)) was patterned via photolithography and etched by ion beam etching (IBE) to serve as an adhesion layer between gold and SU-8 resist (Figure 2f). Etching away the second titanium layer in the previous steps exposes the gold layer underneath. After stripping the AZ ECI resist, a 20 µm of AZ 10XT is spin-coated over the gold layer. The resist was exposed in a mask aligner with a dose of 880 mJ/cm^2^ and developed to define a gold electrode network. Subsequently, the gold electrode network was patterned by etching the gold via IBE (Figure 2g). After etching, the resist was stripped via treatment in oxygen plasma.

### 2.2. Metal-Coated Multi-Layer SU-8 Pillars

After gold electrode patterning, an 8 µm layer of AZ 10XT photoresist was spin-coated on the wafer. The resist was then patterned by photolithography such that the whole wafer, except for the regions where SU-8 will remain after exposure, maintains the positive photoresist layer (Figure S1). A short Descum process (30 s, 200 W) in oxygen plasma was performed to remove any residual photoresist in developed regions.

The 1 mm SU-8 pillars were fabricated by spin-coating multiple layers of SU-8. Before SU-8 coating, the wafers were dehydrated by baking at 90 °C for 15 min. A 300 μm of first SU-8 (SU-8 100, Kayakli Advance Materials, Westborough, MA, USA) layer was spin-coated on the wafer. The wafer was soft baked on a hotplate at 65 °C for 20 min and then at 95 °C for 90 min. The SU-8 layer was exposed on the mask aligner to define 2 mm wide circular pillars (two per microfluidic module), which will later serve as freestanding structures to bias the pseudo-RE. UV exposure was done in proximity contact with 15 µm of exposure gap between the mask and SU-8 layer. The exposure dose of 900 mJ/cm^2^ was delivered in four steps, each of 11.3 s long with 30 s of wait time between each exposure step (Figure S1i–iii). Then, the second layer of SU-8 was spin-coated over the first SU-8 layer, with the same parameters as for the first layer. The second layer was soft baked on a hotplate at 65 °C for 45 min and then at 95 °C for 90 min. The second SU-8 layer was exposed with the same mask and parameters as for the first layer (Figure S1iv–vi). Finally, the third SU-8 layer was spin-coated on top of second the SU-8 layer, with the same parameters as for layers before. The third SU-8 layer was soft baked at 65 °C for 75 min and then at 95 °C for 150 min. The layer was then exposed with the same parameters as for layers before (Figure S1viii–ix). Finally, a post-exposure bake (PEB) for the SU-8 multi-layer structure was performed at 65 °C for 30 min and then at 95 °C for 60 min (Figure S1x).

All three layers were developed simultaneously (Table 1). The wafer was developed face down in a beaker containing PGMEA (Sigma-Aldrich) for 46 min. Throughout the course of development, PGMEA was agitated by gently shaking the beaker in circular motion. After development, the PGMEA was cleaned by IPA rinse and blow-dried under nitrogen. The average height of the SU-8 pillars measured after development was 1012 µm (Figure S1xi).

The SU-8 pillars were then metalized by sputtering a layer of 200 nm gold (Au(2)) through a stencil mask (Figure 2i). The 2 mm wide SU-8 pillars were sputtered through 2.7 mm wide circular openings in the stencil mask. The stencil mask was fabricated from a 4-inch silicon wafer by etching 200 µm deep holes via deep reactive ion etching (Alcatel AMS 200 SE). Thereafter, the wafer backside was grinded down to open the etched holes and thin down the stencil 150 µm. This gold coating ensures an electrical contact between the top of the pillar and the patterned gold electrode lines on the silicon wafer.

### 2.3. Through-PDMS-Vias

The wafer now consists of a patterned gold electrode line with 1 mm high gold-coated SU-8 pillars. To promote adhesion between gold and PDMS, the wafer was silanized with 3-Mercaptopropyl trimethoxysilane (MPTMS). The wafer was dipped in 60 mM MPTMS-ethanol mixture for two hours. Afterwards, the wafer was rinsed in pure ethanol and blow-dried with nitrogen. Before spin coating the wafer with PDMS, the top of the pillars was protected with UV curable tape (Adwill E-6152, Lintec Advanced technologies GmbH, Munich, Germany). The wafer was then exposed to UV light to cure the tape. This reduces the tapes adhesion to the pillar top to minimize shear stress on the pillar during tape removal later. A thin layer of PDMS and curing agent mixture (20:1) was spin coated at 900 rpm over the wafer (Figure 2j). A thick PDMS and curing agent mixture (5:1) was drop casted over a silicon wafer containing negative mold of the pneumatic control valves. Prior to drop casting, the mold wafer was treated with trimethylchlorosilane (TMCS) to facilitate PDMS demolding upon curing. After 15 min of relaxation on a flat surface and degassing under vacuum, both PDMS layers were partially cured at 80 °C for 20 min. Thick PDMS was demolded from the silicon wafer, inlet hole for control valve and 3 mm access holes for SU-8 pillars were punched into it. After dicing, the thick PDMS was manually aligned and placed on the thin spin coated PDMS to be fully cured at 80 °C for 1.5 h (Figure 2k). The curing was done under pressure, applied via weights placed on top of PDMS to remove air bubbles formed during the alignment and bonding of the two PDMS layers.

These metal-coated SU-8 pillars now function as “through-PDMS-vias” for electrical contact to outside world. Lastly, the 500 nm aluminum layer was anodically etched to release the microfluidic module from the wafer (Figure 2l). The wafer was dipped in a 2M sodium chloride (NaCl) solution to act as anode. Another platinum-coated wafer in the NaCl solution functioned as cathode. The anodic dissolution was carried out at constant voltage of 0.7 V until (4–5 h) all microfluidic modules have been released. Upon release, modules were rinsed with DI water and microfluidic inlet/outlets were punched. The microfluidic channel of the module is aligned with the position of ISFETs on CMOS chip (Figure 1b) and reversibly attached by one hour of thermal bonding at 80 °C.

### 2.4. CMOS Chip and Silicon Nanowire ISFETs

To characterize the pseudo-RE, we employed n-type, FDSOI Silicon Nanowire ISFET (length: 2.35 µm and width: 80 nm) with 3 nm thermally grown SiO_2_ as sensing oxide. The ISFETs are 50 nm high and sit on top of a 400 nm thick SiO_2_ insulating bulk oxide layer. The ISFETs are located on a 2 cm × 2 cm CMOS chip. The entire surface of chip, with exception of contact pads and silicon nanowire, is passivated by a multilayer of silicon nitride (50 nm), tetraethyl orthosilicate (300 nm) and phosphosilicate glass (200 nm).

### 2.5. Electrical Characterization

The electrical characterization of metal coated SU-8 pillars and nanowire ISFET in wet conditions were performed by semiconductor parameter analyzer (Agilent 4156C) in Süss PM8 manual probe station. The SPA was controlled by EasyEXPERT group+ (Keysight Technologies, Santa Rosa, CA, USA) software via a GPIB interface. The valve control and electrolyte injection in pseudo-RE module were carried out by pressure system (OB1, Elveflow, Paris, France). The open circuit potential (OCP) measurements for pseudo-RE characterization were carried out by potentiostat (Palmsens2 and PSTrace, Palmsens BV, Houten, The Netherlands).

## 3. Results

### 3.1. Negative Microchannel Mold

**Figure 3:**
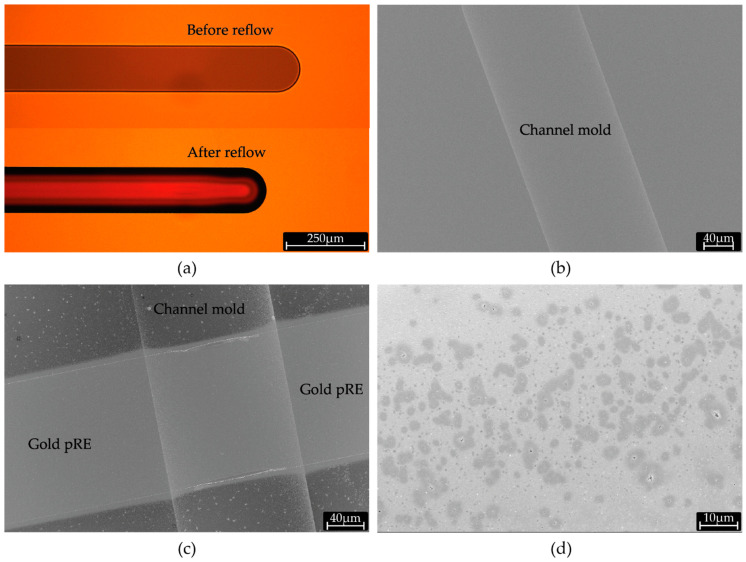
(**a**) Optical micrograph of negative mold for microfluidic channel before and after reflow of AZ 10XT resist. (**b**) SEM image of negative mold after sputtering of aluminum. (**c**) SEM micrograph of a gold pseudo-RE line conformally covering the negative channel mold. (**d**) SEM micrograph after development with KOH based developer, showing dark regions of etched aluminum under the pinholes in the Titanium layer.

A rounded geometry of the channel’s cross section is required for the functioning of the pneumatic valve and, in particular, to allow complete sealing when under pressure from pneumatic valves [7]. A reflow resist (AZ 10XT) was chosen as structural material for the negative mold (Figure 3a). A width of 150 µm is the largest possible channel width whilst maintaining the round cross section. At larger widths, the channel mold’s cross section develops a “cat’s ear” shape upon reflow (Figure S2), which will lead to leaky pneumatic valves. Platinum coating over the mold serves the dual purpose of resist passivation and to later act as an electrical contact layer during anodic dissolution of aluminum (Figure 3b).

### 3.2. Gold Electrode Lines

We had to define specific process parameters for the fabrication of the gold electrode lines in order to ensure conformality of the metal layers over the 12 µm high topology of the mold. Proper definition of gold electrode lines on top of the negative mold was achieved by spin coating a resist with thickness higher than the highest feature on the wafer (Figure 3c). Development of positive resist in photolithography steps after depositing sacrificial aluminum layer was carried out using AZ developer MIC, which unlike TMAH (TetraMethylAmmoniumHydroxide) and KOH based developers does not etch aluminum. Although aluminum layer is covered by titanium (Figure 2b), thin layers tend to have pinholes defects that can allow developer to attack aluminum underneath (Figure 3d).

SU-8 pillars of the foreseen height of 1 mm experience strong shear forces, which can lead to pillars detaching from the wafer. Therefore, a sandwich of titanium adhesion layers is used in regions prone to shear stress in order to enhance adhesion of gold to aluminum and SU-8 to gold (Figure 2c,f). Elsewhere, titanium is etched away by IBE.

### 3.3. Metal Coated SU-8 Pillars

The 1 mm thickness of SU-8 was achieved by multiple SU-8 coatings and exposure steps, followed by a single development step at the end. Although the thickness of the first SU-8 layer upon spin coating is 300 µm, successive coatings of two more SU-8 layers lead to final thickness of ~1000 µm instead of 900 µm. This implies that the second and third coatings are of larger thickness than the first SU-8 layer due to different substrates they get coated on. PEB was performed only on the last coating, since PEB for the exposed layer automatically occurs during soft bake for the next layer. In order to reduce stress build up in the exposed layer during polymerization, slower ramp up and longer hold time at 65 °C were used during soft bake of later coatings of SU-8.

**Figure 4:**
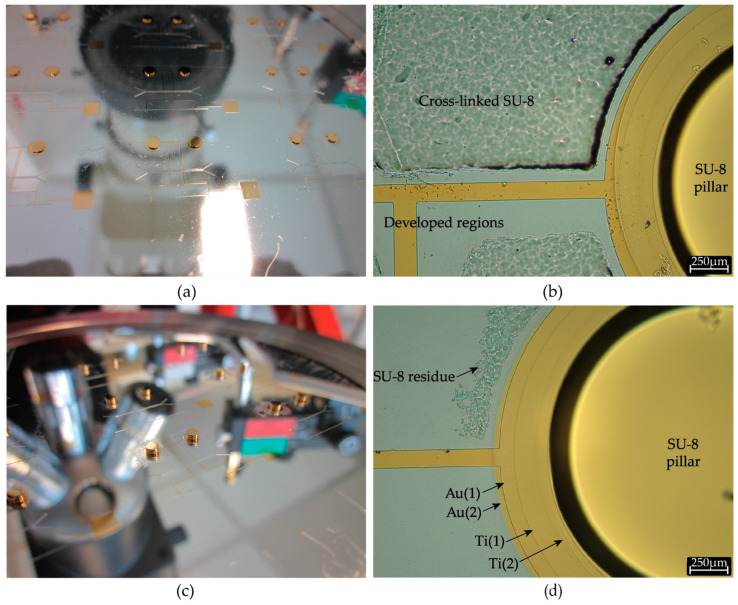
(**a**) Silicon wafer after metallization of 300 µm SU-8 pillars. In this case, no sacrificial resist layer was used prior to SU-8 lithography leading to a thin glossy coating of cross-linked SU-8. (**b**) Optical micrograph of the wafer in Figure 4a, showing unexposed yet cross-linked SU-8 regions resisting overnight development in PGMEA. (**c**) Silicon wafer after metallization of 1 mm SU-8 pillars (resulting from stacking of three SU-8 layers). AZ 10XT was used as sacrificial layer, resulting in vastly cleaner surface after development. (**d**) Optical micrograph of the wafer in Figure 4c, showing the region around the SU-8 pillar. Metallic stack consisting of a sandwich (Ti(1)/Au(1)/Ti(2)/Au(2)) of titanium adhesion and gold electrode layers is also visible.

Baking on hotplate results in non-uniform concentration of solvent in the SU-8 layers, with a highly solvent depleted region closer to the wafer surface. In our case, featuring thick layers and requiring multiple baking steps, it may lead to solvent concentrations in the first SU-8 layer to fall below the recommended processing levels and formation of a thin layer of cross-linked SU-8 above the wafer surface. This thermally cross-linked SU-8 layer cannot be removed during development (Figure 4a,b). The issue of solvent concentration gradient is usually mitigated by skipping the high temperature (95 °C) step and by performing long soft bake solely at low temperatures (for instance 65 °C). However, in case of thick resists, this can extend the soft baking time up to days [8]. Alternatively, we overcame this issue by employing a positive resist (AZ 10XT) as a sacrificial layer, coated prior to SU-8 coating and patterned to remain on the regions were SU-8 was sought to be removed (Figure S1i). AZ 10XT resists only begin to crosslink at 140 °C and therefore is stable at SU-8 soft bake temperatures. This layer is easily removed during development of SU-8 (Figure 4c,d) as PGMEA functions both as SU-8 developer and AZ 10XT solvent. Conventionally, a clean organic-free substrate is recommended to enhance SU-8 adhesion. On the contrary, by deliberately “dirtying” the substrate by coating AZ 10 XT, we prevent adhesion of overbaked/cross-linked SU-8 to the substrate and consequently also improve the development time. Similar use of positive photoresists has been reported previously in sacrificial release process [9] and liftoff of SU-8 structures [10,11]. We employed 8 µm of AZ 10 XT resist to ensure adequate step coverage over the microchannel mold; however, thinner layers can be used on substrates with flatter topography.

**Figure 5:**
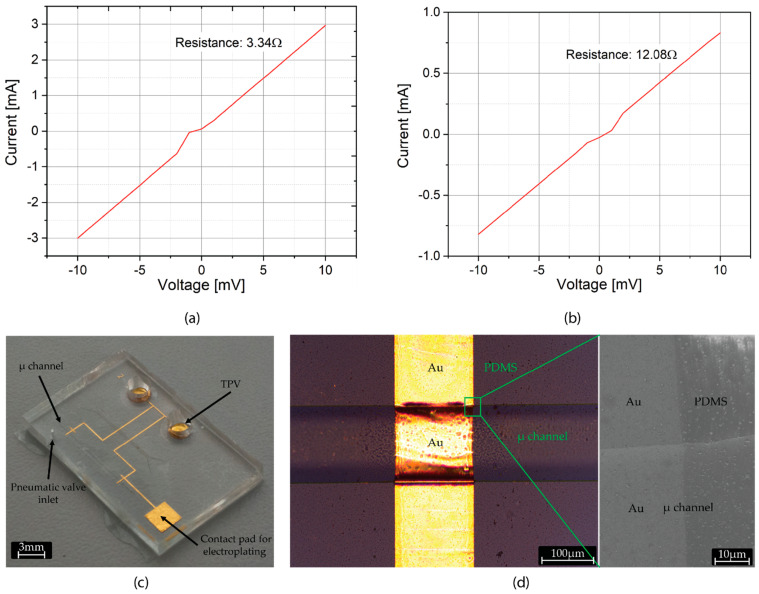
(**a**) Typical two-point resistance measurement between one pair of SU-8 pillars (5 mm apart) before anodic release from wafer. (**b**) Typical two-point resistance measurement between one pair of SU-8 pillars (5 mm apart) after anodic release from wafer. Increase in resistance upon release is due to loss of sacrificial aluminum layer, which provided additional conductive path apart from gold. (**c**) Microfluidic module after sacrificial release, featuring the pseudo-RE network and TPVs. (**d**) Optical (left) and corresponding SEM (right) image of the microchannel showing conformally coated gold pseudo-RE lines in the microchannel. The surface of the pseudo reference electrode exposed to the electrolyte is approximately 150 µm × 100 µm in size, where the exposed length of the electrode is slightly larger than 150 µm due to conformal path in the microchannel.

Metal sputtering of the 1mm high SU-8 freestanding pillars was successfully achieved via stencil lithography. A stencil mask was thinned down to 150 µm by backside grinding to minimize shadow effect of mask sidewalls during gold sputtering. Further thinning was spared to prevent fragility and mask breakage. Conformal coating during sputtering is critical to achieve ohmic electrical contact between the top of the pillar and the planar circular-base on which the pillar is fabricated. The quality of the contacts of the 3D pillars with the wafer was probed by measuring the electrical resistance between the top of the two SU-8 pillars (Figure 5a,b). We estimated an average pillar-to-pillar resistance of 3.51 Ω over 9 different electrode pairs. Moreover, we positively assess the presence of ohmic contact between the 3D pillars in 100% of the fabricated structures (36 3D pillars on two wafers).

### 3.4. Integration of Pseudo-RE in PDMS Microfluidics

Upon metallization of freestanding SU-8 pillars, a pseudo-RE network consisting of planar gold lines and metal-coated SU-8 pillars was integrated in PDMS microfluidics. This tri-dimensional structure has to be transferred to the PDMS microfluidic module that is mold casted over it in order for the microchannel to expose the pseudo-RE on their top internal surface. The transfer is achieved by depositing a self-assembled monolayer of MPTMS in liquid phase on gold surface. The thiol (–SH) end of the silane selectively binds to gold whereas the methoxy (–OCH_3_) end binds to the PDMS, thereby selectively enhancing adhesion of gold to PDMS [12]. Upon sacrificial etch of aluminum layer, the gold pseudo-RE line are conformally transferred onto PDMS microfluidic module (Figure 5c,d). The complete removal of unexposed SU-8 during the earlier development step is a critical requirement at this stage to avoid the residual SU-8 (Figure 4a) to interfere with MPTMS binding on gold. Incomplete removal would result in patchy transfer of gold electrode lines to PDMS and thereby breaking continuity of electrode lines [13]. Moreover, we noticed that upon sacrificial release, this thin residual SU-8 layer transfers to PDMS and greatly reduces the thermal bonding capability of PDMS to CMOS chip.

After spin coating, the PDMS adheres to the sidewall of TPV and forms a meniscus around its base. This results in uneven adhesion between the two PDMS layers in the regions around the TPV and the formation of air bubble. Air can be released by performing the curing under pressure applied via weights. Partially cured PDMS plastically deforms under pressure of the weights, reducing the bubble formation around the SU-8 pillars.

### 3.5. Electrical Characterization of Pseudo-RE and ISFETs

**Figure 6:**
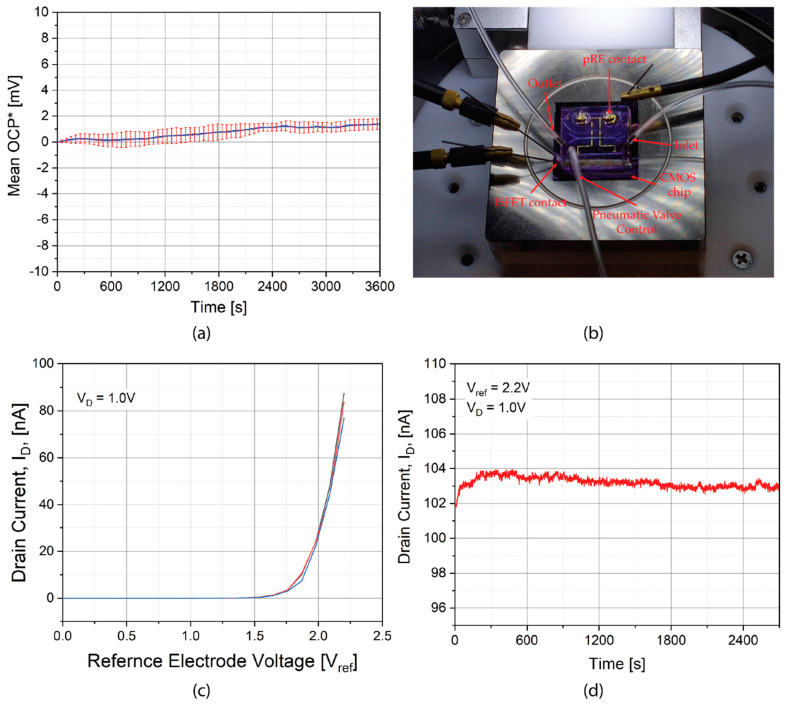
(**a**) Mean OCP (*offset to zero at t = 0) drift of pseudo-RE for three measurements in 3M KCl over a period of an hour. (**b**) Characterization setup showing fluidic conduit, pneumatic valves, and pseudo-RE contact. (**c**) I_D_-V_ref_ characteristics (three repetitions) of electrolytically gated Silicon nanowire ISFET in presence of 3M KCl. (**d**) ISFET static response over time in 3M KCl solution. Estimated drain current drift: ~1 nA/h.

Stability of our pseudo-RE was evaluated by measuring its open circuit potential (OCP) with respect to a commercial flow through Ag/AgCl electrode (16-702, Microelectrodes, Inc., Bedford, NH, USA) over time in a 3M KCl solution (Figure 6a). Observed mean OCP drift of 2 mV/h is similar to other reported gold based miniaturized reference electrodes [14]. Microfluidic module is reversibly coupled via thermal bonding to a CMOS chip (Figure 6b) exposing silicon nanowires to the microfluidic channel. After injection of KCl electrolyte, the nanowire ISFET was gated via an isolated nanoliter sized electrolyte volume, achieved by closing the ends of 1 mm long section of the microchannel by the actuation of pneumatic valves. Figure 6c shows silicon nanowire ISFET’s drain current (I_D_) characteristics under bias (V_ref_) from the pseudo-RE, exhibiting a typical transistor behavior. ISFET demonstrates an excellent static response (I_D_ drift = ~1 nA/h) when under a constant electrode bias applied through the pseudo-RE (Figure 6d).

## 4. Discussion

In this paper, we introduced a new process for pseudo reference electrode (RE) integration in microfluidics based on the combination of a novel SU-8 photolithography process to achieve thick pillar-like vias and a silane-mediated conformal transfer of metal electrodes into PDMS microfluidics. The 3D conformal design of pseudo-RE when combined with parcellization microfluidics allows very small analyte volumes to be isolated and characterized by ISFET. Small reaction volumes benefit from increased sensitivity, less contaminations and reduced cost. The pseudo-RE design here presented is scalable to a large number of reaction parcels, each of which can be independently monitored by ISFET or any other solid-state sensor, thereby increasing the throughput. The density of reaction parcels is only limited by photolithography of the mold to form the microfluidic module. Our design synergizes the advantages of low reaction volumes with unprecedented pH resolution and CMOS scalability of ISFETs. The approach is most suitable for label-free, semiconductor based molecular diagnostic applications that require sample digitization. For example, digital molecular assays for early detection of sepsis causing pathogens [15,16] have been shown to have significantly reduced time to detection when compared to blood culture based identification, leading to reduced mortality rates. Similar digital assays have been used in absolute quantification of nucleic acids [17], digital-ELISA [18], tumor-DNA analysis [19], etc. Hassibi et al. have demonstrated multiplexed detection of nucleic acid amplification and sequence analysis on CMOS chip consisting of 32 × 32 array of photodiodes using fluorescence detection [20]. However, requirement of external optical excitation source, utilization of post-CMOS processing to integrate optical filter layers and capture probes for detection of DNA increases the complexity of the biosensor device. Leveraging scaling advantage of CMOS technology and electrochemical sensing of molecular assays, optical readout in these applications can be replaced by label-free electronic readout via ISFETs, leading to increased readout speeds and reduced device footprint. Use of segregated volumes for amplification reaction and pH-based amplification assays can obviate the need for post-CMOS processing and complex capture probes.

A key challenge in implementation of large number of segregated biochemical reactions in ultra-low volume chambers lies in the evaporation of water from biochemical reactions due to elevated operating temperatures, high surface-to-volume ratio of reaction chambers, and gas permeability of PDMS. This issue can generally be tackled by integration of low-permeability polymer layers in PDMS microfluidics [21,22].

### 4.1. Conformal Pseudo Reference Electrode

Miniaturized reference electrodes (mRE) have been increasingly used to develop integrated electrochemical biosensors for various applications including POC scenarios. General approaches to fabrication of mRE involve post-processing of the chip [23] or PCBs [24] to co-integrate them with the sensing sites. These approaches are generally ad-hoc and lack scalability. Conformal integration of the electrode on the inside of the microchannel allows our pseudo-RE design to be easily scaled to any number just by changing the photolithography mask. When combined with partitioning microfluidics, we can simultaneously sense multiple ultra-low segregated volumes. Norian et al. have demonstrated on-chip PCR in 1.2 nL volumes, using integrated photodiodes [25]. Here, we have performed non-optical chemical sensing via solid-state device in the smallest parcellized volume ever demonstrated. The presented pseudo-RE design can be easily converted to Ag/AgCl reference electrode system by electrodeposition of silver and its subsequent chlorination [26]. We believe that our design can solve the issue of short lifetime of miniaturized Ag/AgCl electrodes resulting from degradation of AgCl in aqueous solutions [27]. Few hundred nanometers of AgCl layer can easily be consumed in constant-flow conditions or big electrolyte volumes. Conversely, due to the ultra-low volume of the electrolyte in our design, the concentration of Cl^-^ ions in the electrolyte will reach equilibrium with minimal loss of AgCl layer.

In general, micro-fabricated electrodes, when used in large chambers, suffer from poor stability [28]. In this paper, we could benefit from the high surface-to-volume ratio of pseudo-RE in small volume chambers, resulting in a low (~1 nA/h) drain current drift (Figure 6d). On the other side, the OCP drift reported in Figure 6a may not reflect the true behavior of pseudo-RE in no-flow ultra-low volume conditions as OCP characterization cannot be performed in ultra-low volumes due to the size of standard reference electrode.

### 4.2. Through-PDMS-Vias

The need to have reference electrode network integrated in the fluidics necessitates the employment of TPVs in the microfluidic module. As the reference electrode network lies on the lower face of the microfluidic module, the only way to electrically access it by use of vias. Metal-coated SU-8 pillars can be employed as TPV of variable heights. The aspect ratio of the TPV depends on SU-8 processing, which can achieve aspect ratios of 20:1 and heights greater than 1.5 mm [29] on silicon substrate. SU-8 features heights of up to 7 mm on glass substrates have been demonstrated by using back side UV exposure [30]. However, backside exposure for SU-8 lithography cannot be used if the transparent substrate already had opaque features that interfere with exposure mask, as is the case with our process. Carbon nanotube (CNT) forest-based scaffolding structures have been demonstrated as TPV for application in 3D stacking of flexible electronics [31]. However, the high temperature required for CNT growth puts thermal budget constraints on prior process steps. SU-8 based TPV circumvents the said process limitations and provides a better alternative to CNT-based TPV, especially for biological applications thanks to SU-8′s biocompatibility [32].

Metal coated SU-8 pillars have been demonstrated as vertical electrodes with electrical continuity between the pillar side wall and the planar bottom electrodes [33]. Use of stencil lithography for metal deposition does impose limits on miniaturization and density of TPV. Our TPV strategy also offers improved access to electrode networks in applications like wearable microfluidics and flexible surface implants [34], where the conformality of flexible electronics to tissue is of critical importance.

### 4.3. SU-8 Processing

We propose a new method for SU-8 that reduces processing time and provides better substrate quality after development, especially for very thick SU-8 layers. Due to long baking times and non-uniform solvent concentration in thick SU-8 layers, a thin SU-8 layer in proximity to wafer surface gets partially cross-linked and therefore undevelopable. It is critical to have SU-8 residue free substrate after development for reliable silane mediated bonding between pseudo-RE network and PDMS. Utilization of a thin layer of positive photoresist offers multiple processing advantages. As thin photoresist layer discourages SU-8 adhesion, soft bake time can be further reduced by baking at higher (>95 °C) temperatures [29], without being concerned of overbaking SU-8. Although development time is highly dependent on operating conditions, use of positive photoresist layer led to almost 50% reduction in development time for 300 µm SU-8 layer. In the current work as resolution of SU-8 features was of less concern, hard baking of positive resist was not performed prior to SU-8 coating. However, upon SU-8 coating solvent from SU-8 will mix with the positive resist, which may result in lateral flow of the positive resist into SU-8 exposure regions, leading to loss of resolution. To mitigate it, a hard-baked thin positive resist layer is highly recommended prior to SU-8 coating.

## Figures and Tables

**Table 1:** Fabrication protocol for 1 mm high pillars using three SU-8 layers and a single development.

SU-8 Layer	Spin Coat	Soft Bake	Exposure	PEB	Development
First Layer	5 s ramp up to 500 rpm 10 s hold at 500 rpm 3.3 s ramp up to 1000 rpm 28 s hold at 1000 rpm 1 s at 14,000 rpm 5 s hold at 1000 rpm	3200 s hold at 30 °C 700 s ramp up to 65 °C 1800 s hold at 65 °C 700 s ramp up to 95 °C 5400 s hold at 95 °C Cool down to RT	Type: Proximity Exposure gap: 15 μm Duration:4 × 11.3 s, 30 s wait Intensity: 20 mJ/cm^2^	Occurs during soft bake of second layer	No development
Second Layer	5 s ramp up to 500 rpm 10 s hold at 500 rpm 3.3 s ramp up to 1000 rpm 28 s hold at 1000 rpm 1 s at 14,000 rpm 5 s hold at 1000 rpm	3200 s hold at 30 °C 2100 s ramp up to 65 °C 2700 s hold at 65 °C 1800 s ramp up to 95 °C 5400 s hold at 95 °C Cool down to RT	Type: Proximity Exposure gap: 15 μm Duration:4 × 11.3 s, 30 s wait Intensity: 20 mJ/cm^2^	Occurs during soft bake of third Layer	No development
Third Layer	5 s ramp up to 500 rpm 10 s hold at 500 rpm 3.3 s ramp up to 1000 rpm 28 s hold at 1000 rpm 1 s at 14,000 rpm 5 s hold at 1000 rpm	3200 s hold at 30 °C 2100 s ramp up to 65 °C 2700 s hold at 65 °C 1800 s ramp up to 95 °C 5400 s hold at 95 °C Cool down to RT	Type: Proximity Exposure gap: 15 μm Duration:4 × 11.3 s, 30 s wait Intensity: 20 mJ/cm^2^	10 s hold at 30 °C 1800 s ramp up to 65 °C 1800 s hold at 65 °C 1800 s ramp up to 95 °C 3600 s hold at 95 °C Cool down to RT	46 min in PGMEA

## Supplementary Materials

The following are available online at https://www.mdpi.com/article/10.3390/mi12070762/s1. Figure S1: Process flow steps for multi-layer SU-8 lithography. Figure S2: Height and cross-section profile after reflow of the of microfluidic mold for different width: (a) 20 µm; (b) 100 µm; (c) 150 µm; (d) 200 µm. “Cat’s ear” profile for seen for mold width larger than 150 µm. Figure S3: (a) 3D schematic of microfluidic module mounted on the CMOS chip. Thick PDMS is not shown for sake of clarity. (b) Top view and side view of the microfluidic module without the thick PDMS layer. (c) Top and side view of the microfluidic module with thick PDMS containing the pneumatic valves.
