# Supplementary material for: Integration of Ultra-Low Volume Pneumatic Microfluidics with a Three-Dimensional Electrode Network for On-Chip Biochemical Sensing
Source: Micromachines (Basel). 2021 Jun 28;12(7):762. doi: 10.3390/mi12070762 (PMC8306664; doi:10.3390/mi12070762)
Supplement: Supplementary file 1 [file micromachines-12-00762-s001.zip › micromachines-1263347-supplementary.pdf]

# Supplementary Materials

# Integration of Ultra-Low Volume Pneumatic Microfluidics with a Three-Dimensional Electrode Network for On-Chip Biochemical Sensing

**Saurabh Tomar, Charlotte Lasne, Sylvain Barraud, Thomas Ernst and Carlotta Guiducci**

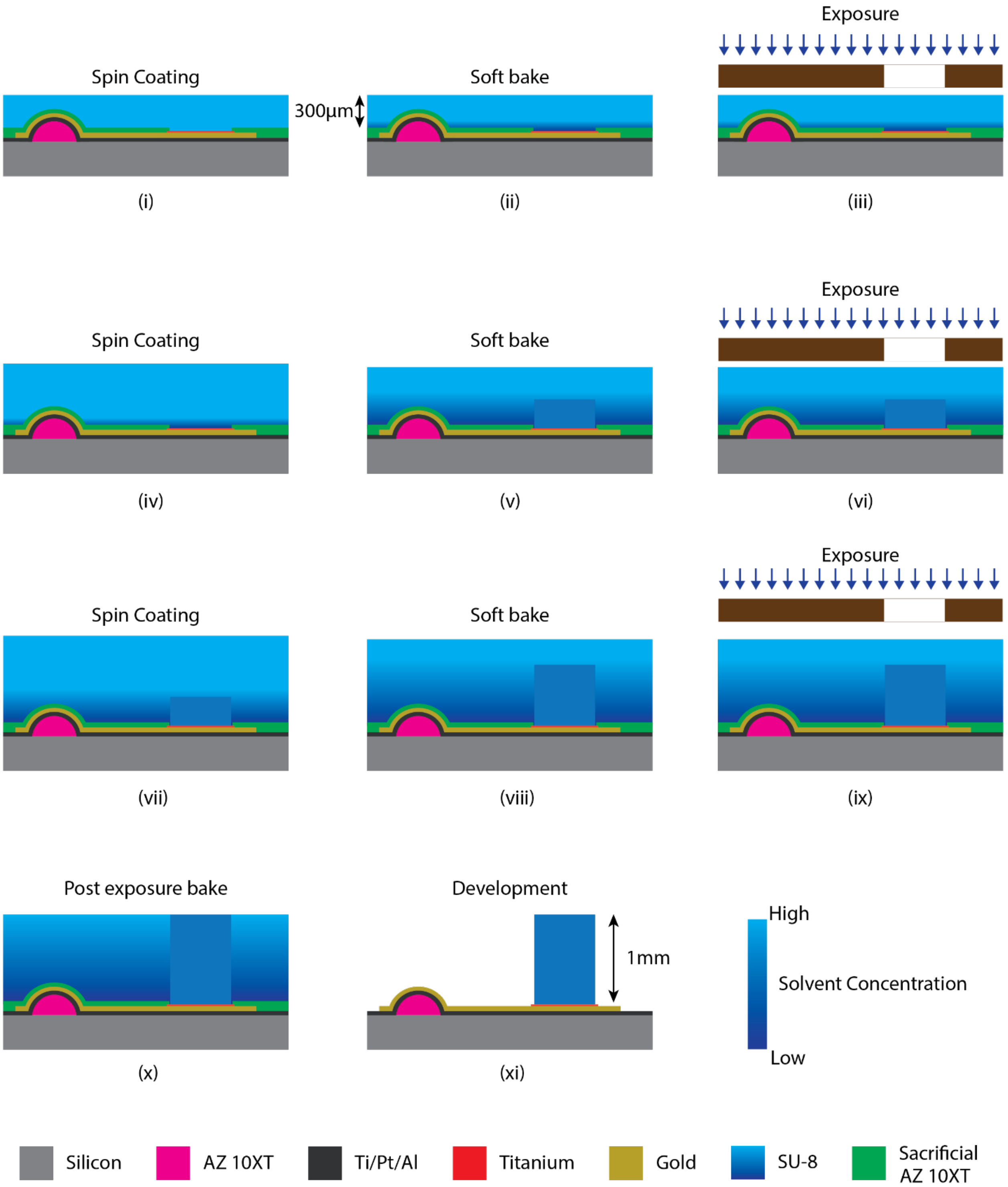


**Figure S1**. Process flow steps for multi-layer SU-8 lithography.

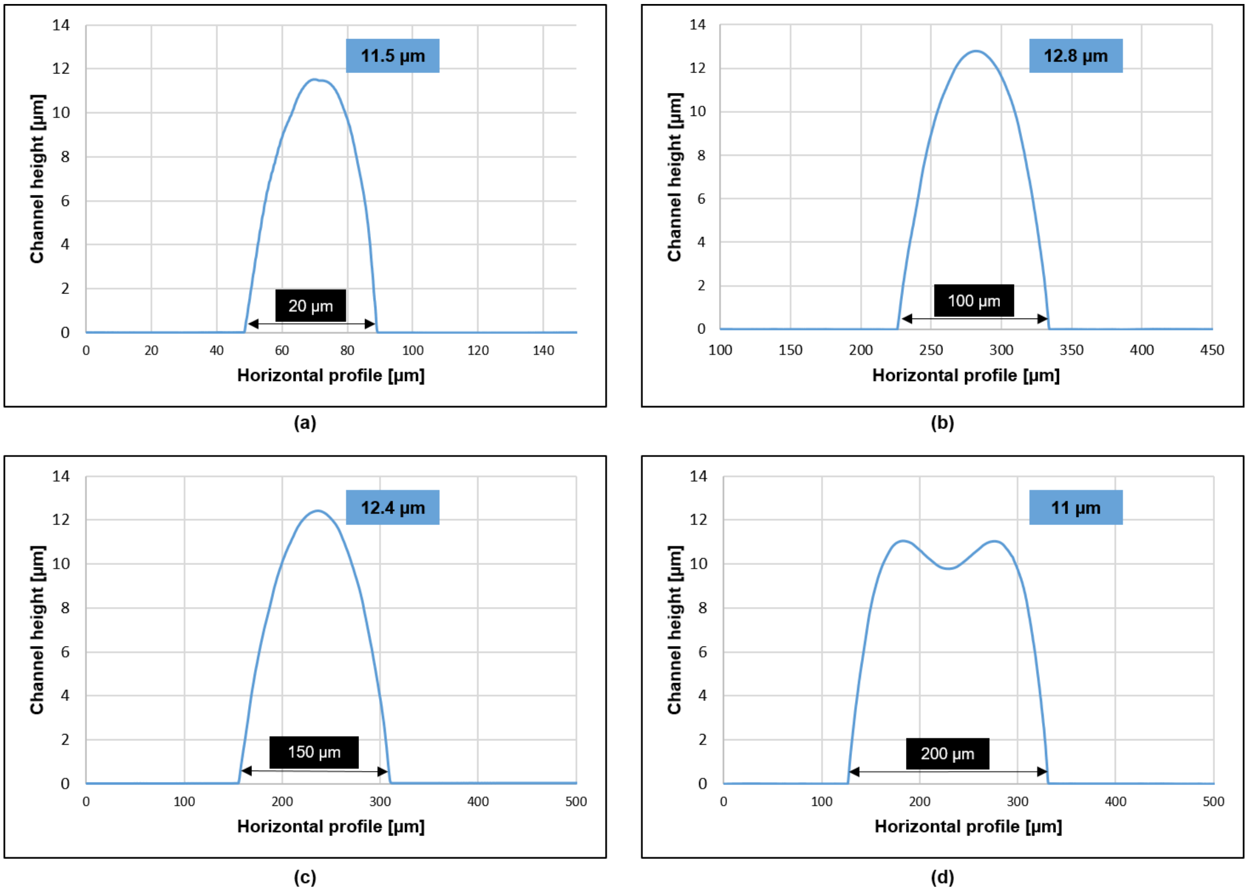


**Figure S2**. Height and cross-section profile after reflow of the of microfluidic mold for different width: (a) 20 µm; (b) 100 µm; (c) 150 µm; (d) 200 µm. "Cat's ear" profile for seen for mold width larger than 150 µm.

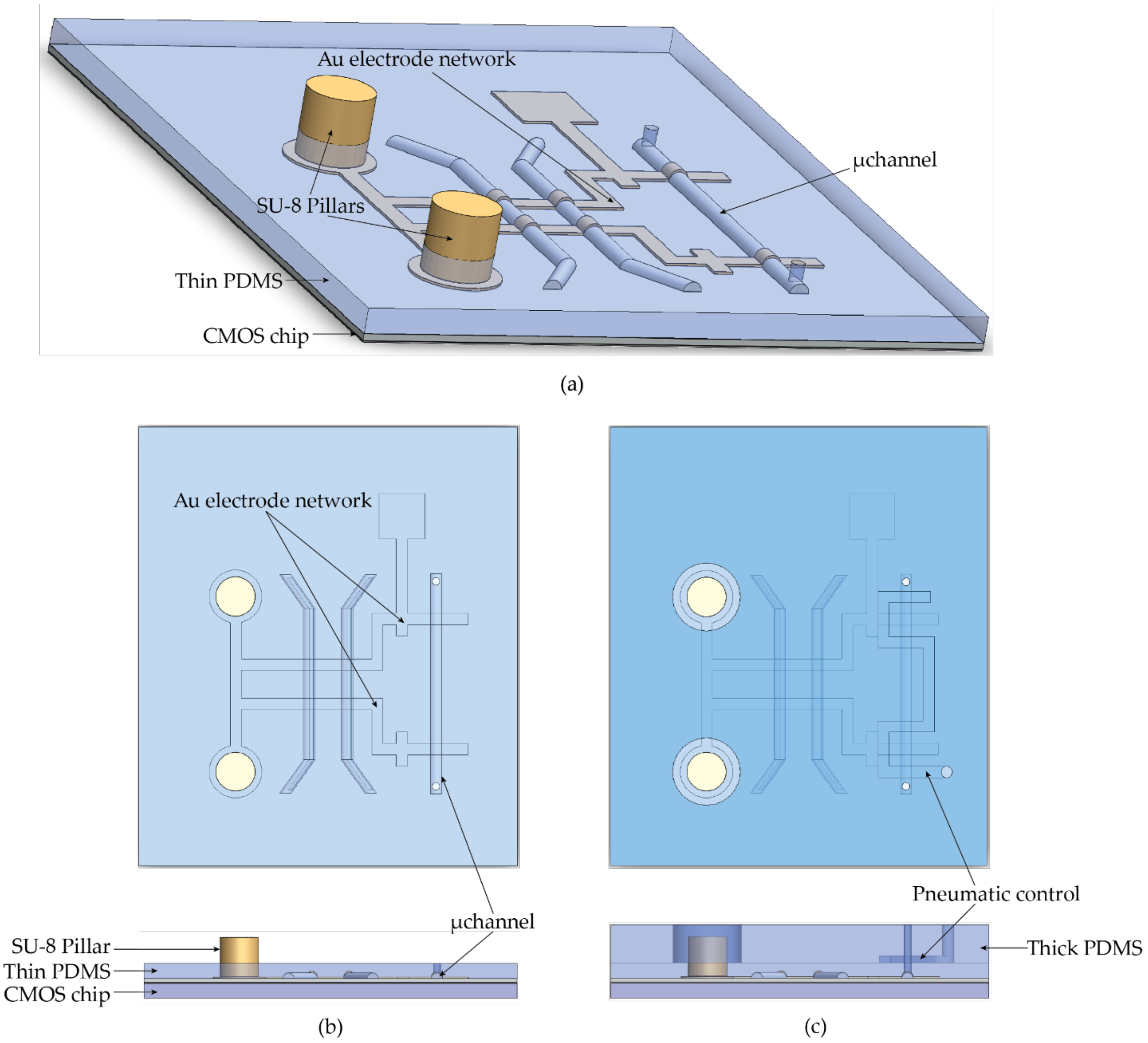


**Figure S3**: (a) 3D schematic of microfluidic module mounted on the CMOS chip. Thick PDMS is not shown for sake of clarity. (b) Top view and side view of the microfluidic module without the thick PDMS layer. (c) Top and side view of the microfluidic module with thick PDMS containing the pneumatic valves.
